# Genetic Variation and Heritability for Hydrogen Cyanide in Fresh Cassava Roots: Implications for Low-Cyanide Cassava Breeding

**DOI:** 10.3390/plants13091186

**Published:** 2024-04-24

**Authors:** Michael Kanaabi, Mukasa B. Settumba, Ephraim Nuwamanya, Nicholas Muhumuza, Paula Iragaba, Alfred Ozimati, Fatumah B. Namakula, Ismail S. Kayondo, Julius K. Baguma, Ann Ritah Nanyonjo, Williams Esuma, Robert S. Kawuki

**Affiliations:** 1 College of Agricultural and Environmental Sciences, Makerere University (MAK), Kampala P.O. Box 7062, Uganda; sbmukasa@caes.mak.ac.ug (M.B.S.); ephraim.nuwamanya@mak.ac.ug (E.N.); nicholasmuhumuza1@gmail.com (N.M.); ozimatialfred@gmail.com (A.O.); bagumakj@gmail.com (J.K.B.);; 2 National Crops Resources Research Institute (NaCRRI), Kampala P.O. Box 7084, Uganda; iragapaula@gmail.com (P.I.); fatumahb7@gmail.com (F.B.N.); kawukisezirobert@gmail.com (R.S.K.); 3 College of Natural Sciences, Makerere University (MAK), Kampala P.O. Box 7062, Uganda; 4 International Institute for Tropical Agriculture (IITA), Ibadan 200113, Nigeria; s.kayondo@cgiar.org

**Keywords:** breeding efficiency, end user preference, phenotyping, selection, genotype by environment interaction

## Abstract

Breeding for low-hydrogen-cyanide (HCN) varieties is a major objective of programs targeting boiled cassava food products. To enhance the breeding of low-HCN varieties, knowledge of genetic variation and trait heritability is essential. In this study, 64 cassava clones were established across four locations and evaluated for HCN using three HCN assessment methods: one with a 1 to 9 scale, on with a 0 ppm to 800 ppm scale, and a quantitative assay based on spectrophotometer readings (HCN_Spec). Data were also collected on the weather variables precipitation, relative humidity, and temperature. Highly significant differences were observed among clones (*p* < 0.001) and locations (*p* < 0.001). There was also significant clone–environment interactions, varying from *p* < 0.05 to *p* < 0.001. Locations Arua and Serere showed higher HCN scores among clones and were associated with significantly higher (*p* < 0.001) mean daily temperatures (K) and lower relative humidity values (%) across 12 h and 18 h intervals. Within locations, HCN broad sense heritability estimates ranged from 0.22 to 0.64, while combined location heritability estimates ranged from 0.14 to 0.32. Relationships between the methods were positive and strong (r = 0.75–0.92). The 1 to 9 scale is more accurate and more reproducible than either the 0 to 800 ppm scale or spectrophotometric methods. It is expected that the information herein will accelerate efforts towards breeding for low-HCN cassava varieties.

## 1. Introduction

Cassava (*Manihot esculenta* Crantz) is a staple for more than 600 million people in Eastern, Western, and Southern Africa [1]. These regions produce and consume more than 54% of the world’s cassava, underpinning the importance of the crop to their livelihoods. The crop’s clonal nature, ability to survive in marginal soils, tolerance to drought [2], and perennial nature, which allows for peace meal harvest, allow for a diversity of its uses for food, feed, and industry, making it an important food security crop [3,4,5]. However, cassava’s usefulness is limited by the presence of cyanogenic glucosides, namely linamarin and lotaustralin, in the leaves and roots [6,7]. Linamarine readily hydrolyzes into glucose and acetone cyanohydrin in the presence of linamarase enzyme and in a neutral or alkaline medium, while acetone cyanohydrin decomposes to liberate cyanide and a hydrogen ion [8].

The production of hydrogen cyanide (HCN) in plants is said to be a defense mechanism against herbivores and pathogens [9,10]. Total cyanogenic concentrations in cassava may vary with the cultivar, environmental conditions, cultural practices, and plant age. Indeed, a HCN content in the range of 2 ppm to >1000 ppm has been reported [8,11]. Cassava is thus classified as “sweet” if it has a root HCN content < 100 ppm on a wet basis and “bitter” if it has root HCN > 100 ppm [12,13]. In fact, cassava with a HCN content > 10 ppm on a dry weight basis or >100 ppm on a fresh-weight basis is considered not safe for human consumption and should undergo further processing before consumption [14].

A dietary intake of high amounts of HCN can be toxic and/or lethal [15,16]. Prolonged dietary exposure to HCN is linked to degenerative conditions of the nervous system, and konzo and tropical ataxic neuropathy [17,18,19]. Despite these risks, some communities still cultivate bitter (high HCN) varieties because they are not stolen by humans or rodents [20]. The rate of adoption of new crop varieties is dependent upon end user preferences [21,22,23,24].

Consumers of boiled cassava have a preference for non-bitter cassava varieties [3,25,26], characterized by low HCN content. HCN content is thus a major component of cassava root quality for boiled or steamed cassava roots, a product that is desired by consumers of cassava. In the Amazon region, farmers readily classify cassava varieties into sweet, i.e., aipim or macaxera, table varieties, or bitter varieties, which are said to be suitable for industry [27], while in Malawi, farmers classify their varieties as either “cool” or “bitter” depending on HCN levels [28]. Owing to the increasing interest in boiled cassava, efforts are underway to initiate breeding for low-HCN cassava varieties. However, the success of these efforts largely depends on the amount of variation present and the proportion of this variation that is heritable [28,29]. In addition to genotypic differences, variation in fresh cassava root HCN content is said to be influenced by the soil nutrient status [30] and climatic factors such as precipitation and temperature [31].

Phenotyping for the HCN content of fresh cassava roots in cassava breeding programs is commonly performed using any of the three variations of the picrate method. These include a 0 to 800 ppm color scale [8], the modified picrate method [6,32,33], which allows for the collection of semi-quantitative data using a spectrophotometer, and another modification of the picrate method suggested by Fukuda et al. [34], which follows a 1 to 9 color scale. All these methods have ontologies for data handling in the cassava breeding database, Cassavabase [35], and are thus deployed in major cassava breeding programs. Whether or not these methods provide comparable results is unknown. Furthermore, it is not known whether or not sufficient genetic variation exists in available germplasm to initiate long-term breeding for low-HCN cassava. The effect of weather variables on HCN phenotypic expression within the available germplasm also remains unknown. Thus, in this study, we sought to (a) assess the extent of genetic variation and heritability for HCN within the study germplasm; (b) compare the accuracy of the three HCN phenotyping protocols; (c) assess the relationship between the weather variables precipitation, temperature, and relative humidity, and fresh cassava root HCN phenotypic expression.

## 2. Results

### 2.1. Variability for HCN in Fresh Cassava Roots

Highly significant differences among clones (*p* < 0.001) and locations (*p* < 0.001) were observed under all three HCN assessment methods. Furthermore, there were significant clone–environment interactions for the three methods (Table 1).

On the 1–9_Scale, fresh root HCN content ranged from 2 to 9 with a combined location average of 6.56. The highest average HCN content was recorded in Arua (7.70) followed by Serere (7.58), while Namulonge (5.45) had the lowest average HCN content (Table 2). On the 0 to 800 scale, fresh root HCN content ranged from 65 ppm to 800 ppm with a combined location average of 430.21 ppm. The highest average HCN content (614.24 ppm) was again recorded in Arua, while the lowest (236.03 ppm) was again recorded in Namulonge (Table 2). For the HCN_Spec method, fresh root HCN content ranged from 119.6 ppm to 676.3 ppm with a combined location average of 504.1 ppm. However, the highest average fresh root HCN content (608.53 ppm) was recorded in Serere while the lowest (399.39 ppm) was recorded in Tororo. Generally, HCN observations at Namulonge were comparable to those at Tororo while observations for Serere were comparable to those at Arua (Table 2).

Within sites, broad sense heritability ranged from 0.46 to 0.54 on the 1 to 9 scale, 0.24 to 0.64 on the 0 to 800 ppm scale, and 0.22 to 0.56 using the spectrophotometric method. Across locations, broad sense heritability was 0.14 for the spectrophotometric method, 0.26 for the 1 to 9 scale, and 0.32 for the 0 to 800 ppm scale. The genetic component of the variation (V_g_) was 0.48 for the 1 to 9 scale, 3596 for the spectrophotometric method, and 13,990 for the 0 to 800 ppm scale (Table 2).

**Figure 1:**
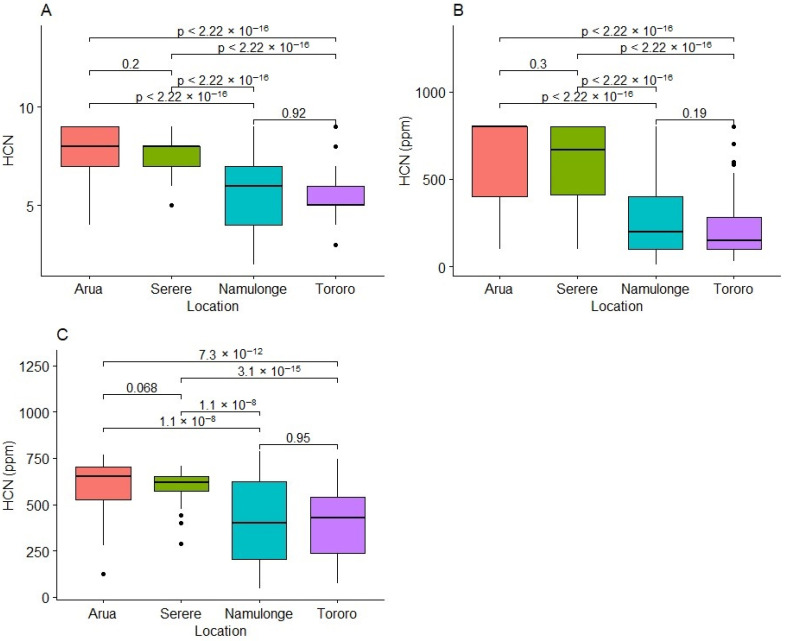
Variation in fresh cassava root HCN content across test sites based on three assessment methods: the 1 to 9 categorical scale (**A**), the 0 to 800 ppm categorical scale (**B**), and the spectrophotometric method (**C**).

On the 1–9_Scale, Arua had clones scoring 4 to 9 with a high median value of 8. The distribution was skewed upwards (Figure 1A). In Serere, scores ranged from 3 to 9, with 50% of the clones scoring between 7 and 8, thereby skewing the distribution upwards. In Tororo, HCN scores ranged from 3 to 9, with 50% clones scoring between 5 and 6, while in Namulonge, scores ranged from 2 to 9, with a median of 6 and a near-uniform distribution of scores across the scale (Figure 1A). However, for the 0–800_Scale, HCN scores ranged from 100 to 800 ppm in Arua with 75% clones scoring 400–800 ppm, thereby skewing the distribution upwards. Similarly, in Serere, HCN scores ranged from 100 to 800 ppm, with a median score slightly below 700 ppm. The distribution of scores was skewed upwards. In Namulonge and Tororo, the median HCN scores were 200 ppm and 150 ppm, respectively, with scores ranging from less than 50 to 800 ppm. For both locations, the distribution was skewed downwards (Figure 1B). A similar trend was observed using the spectrophotometric method (Figure 1C). Generally, Arua and Serere had high HCN values, thereby skewing the distributions upwards, while Namulonge and Tororo had nearly uniform distributions (Figure 1).

### 2.2. Accession Ranking for Fresh Cassava Root HCN Content Using BLUPS

BLUPS consistently ranked four of the five bitter local farmers’ clones among the top 10 for high HCN content (Table 3). Over all, six clones, Tongolo, Nyamatia, Kazimwenge, Quinine, UG16F303P006, and UG16F158P004, were consistently ranked among the top 10 for high HCN content. On the other hand, five clones, UG16F290P295, UG16F293P169, UG16F057P001, UG16F001P013, and UG16F290P075 were consistently ranked among the top ten for low HCN content. Surprisingly, the local bitter farmers’ accession Edwarat was ranked among the top 10 low-HCN clones both on the 1 to 9 scale and under spectrophotometric methods. Overall, five clones were consistently selected under all methods, six clones on the 1 to 9 scale and the 0 to 800 scale, and nine clones on the 1 to 9 scale and under spectrophotometric method (Table 3).

### 2.3. Comparison of Phenotyping Method Performance

#### 2.3.1. Correlation and Regression between the Methods

**Figure 2:**
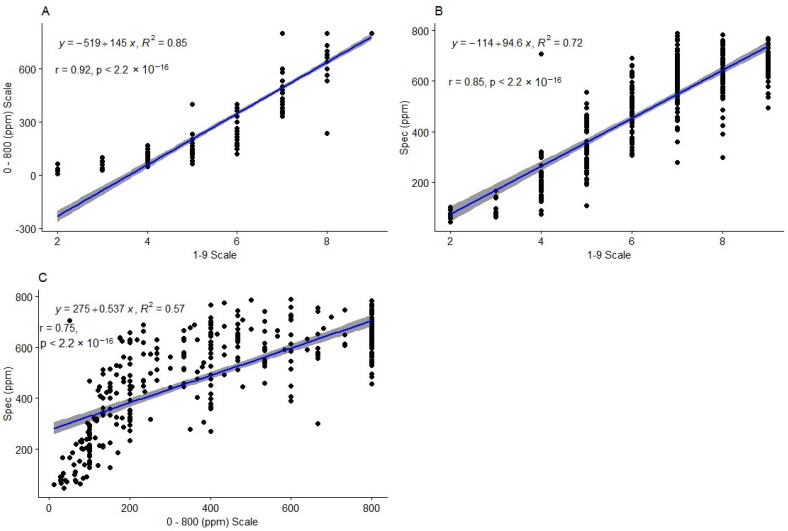
Scatter plots with regression lines and confidence intervals, the regression equations and correlation coefficients depicting the relationship between the HCN phenotyping methods based on the picrate protocol. The relationship between the 1 to 9 scale and the 0 to 800 ppm scale is shown in plot (**A**). Plot (**B**) shows the relationship between the 1 to 9 scale and the spectrophotometric method, while plot (**C**) shows the relationship between the 0 to 800 ppm scale and the spectrophotometric method. ppm = parts per million; R^2^ = coefficient of determination; r = correlation coefficient; Spec = spectrophotometric method.

Correlation analysis revealed a strong positive relationship between all the methods (Figure 2). The highest correlation coefficient (r = 0.92, *p* < 0.001) was observed between the 1 to 9 scale and the 0 to 800 ppm scale (Figure 2A). Furthermore, there was a strong positive relationship (r^2^ = 0.85, *p* < 0.001) between the 1 to 9 scale and the spectrophotometric method (Figure 2B), and between the 0 to 800 ppm scale and the spectrophotometric method (r^2^ = 0.75, *p* < 0.001) (Figure 2C).

The highest coefficient of determination (R^2^ = 0.85) was observed between the 1 to 9 scale and the 0 to 800 ppm scale. The relationship between the two methods is represented by the regression equation y=−519+145x (Figure 2A). The coefficient of determination between the 1 to 9 scale and the spectrophotometric method was also high (R^2^ = 0.72). The relation between the two methods is summarized by the regression equation y=−114+94.6x (Figure 2B). On the other hand, the coefficient of determination between the 0 to 800 ppm scale and the spectrophotometric method (R^2^ = 0.57) was weak. The relation between these two methods is summarized by the regression equation y=275+0.537x (Figure 2C).

#### 2.3.2. Comparison of Residuals

**Figure 3:**
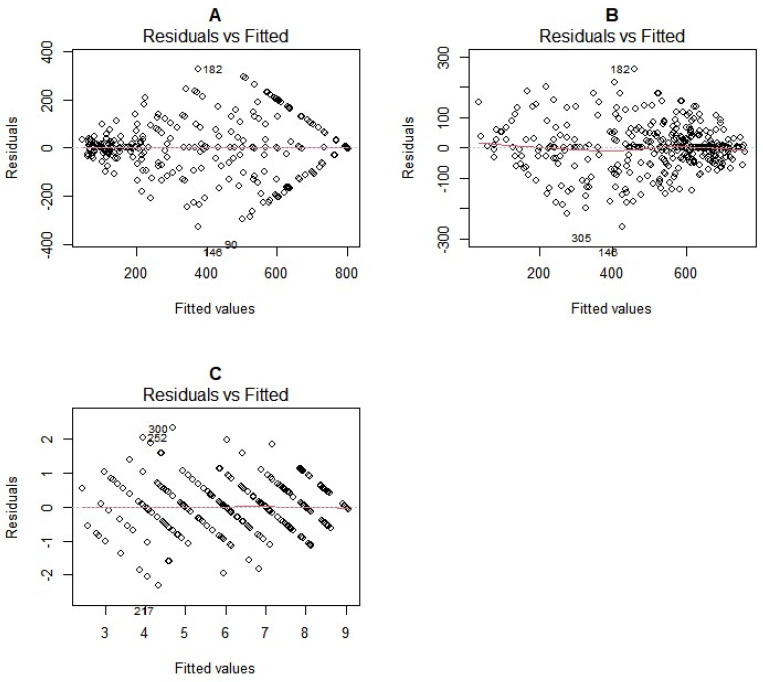
Residual vs. fitted plots for the 0 to 800 ppm scale (**A**), spectrophotometric method (**B**), and 1 to 9 scale (**C**).

Large residuals were observed between 300 and 600 ppm for the 0 to 800 ppm scale (Figure 3A) and between 200 and 400 ppm for the spectrophotometric method (Figure 3B). Residuals were more clustered towards the zero line for higher HCN readings (more than 400 ppm) for the spectrophotometric method, while for the 0 to 800 ppm scale, this pattern was observed for lower HCN scores (less than 200 ppm). There was a random distribution of residuals for the 1 to 9 scale (Figure 3C).

For a uniform comparison of residuals for all three methods, we computed the relative standard error (RSE). For the 1 to 9 scale, the RSE was 17.4; the RSE was 25.7% for the Spec method and 41.32% for the 0 to 800 scale method, indicating that the 1 to 9 scale was the most accurate, followed by the spectrophotometric method, with the 0 to 800 scale being least accurate.

### 2.4. Variation in Weather Variables at Experimental Test Locations

**Figure 4:**
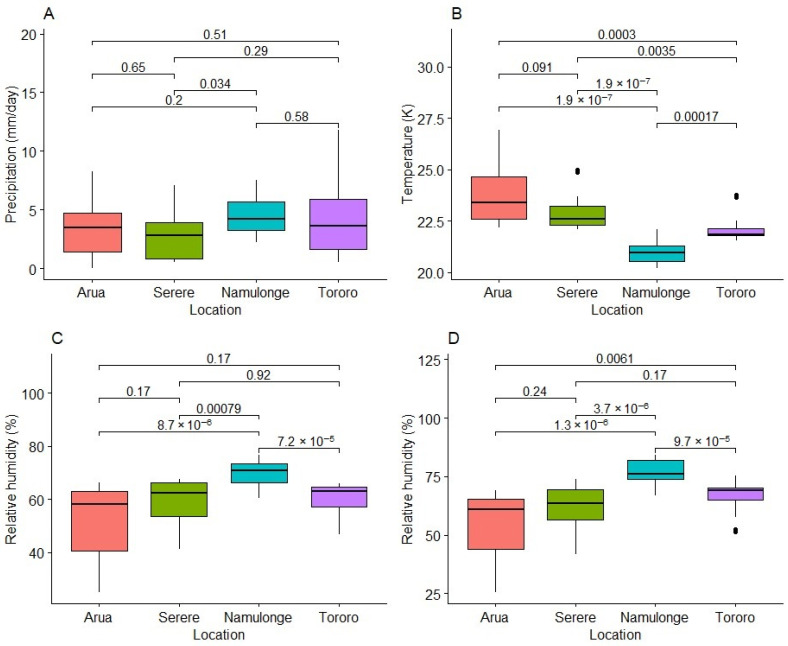
Variation in the weather variables precipitation (**A**), temperature (**B**), relative humidity at 12 h (**C**), and relative humidity at 18 h (**D**) among the test locations. The Kruskal–Wallis test was used to show significance or no significance in the observed differences of weather parameters at α = 0.05 across locations. % = percentage; K = Kelvin; mm/day = millimeters per day.

We compared locations for differences in the amount of precipitation (mm/day), average daily temperature (K), and relative humidity (%) at 12- and 18 h intervals. There was significant variation (*p* < 0.001) among locations for 24 h temperature, relative humidity at 12 h, and relative humidity at 18 h (Figure 4). Except in terms of precipitation, Namulonge was significantly different from all other locations. Namulonge received significantly higher precipitation (*p* = 0.034) than Serere but not was significantly different from Tororo and Arua (Figure 4A). Namulonge had the lowest average 24 h temperature (20.97 K) followed by Tororo (22.16 K), Serere (22.99 K), and Arua (23.90 K). Arua was significantly hotter than Tororo (*p* = 0.0003) but did not differ from Serere in terms of temperature (Figure 4B). Relative humidity at 12 h for Namulonge was high (average = 70%) and significantly different from that at all other locations. However, there were no significant differences between the other locations (Figure 4C). At 18 h, Namulonge had the highest relative humidity (77.03%) and was significantly different from other locations in terms of this. It was followed by Tororo (66.17%) and Serere (60.98%). Arua, with the lowest relative humidity (54.53%), was not significantly different from Serere. Serere was also not significantly different from Tororo, but Tororo was significantly different from Arua (*p* = 0.0061) (Figure 4D) in terms of this.

## 3. Discussion

### 3.1. Genetic Variability and Heritability for HCN

A major objective of this study was to establish whether significant genetic variation exists for HCN in available germplasm, and whether different assessment methods vary in precision. Highly significant variation for HCN among clones was observed. Fresh root HCN content ranged from 2.5 to 9 with a mean of 6.56 on the 1 to 9 scale, 65.2 to 800 ppm on the 0 to 800 ppm scale with a mean of 430.21, and 119.60 to 676.30 ppm with an average of 504.10 ppm using HCN_Spec. It is thus evident that there was variability for HCN within the evaluated germplasm. The evaluated germplasm comprised both local landraces and elite clones. This favors selection for low HCN. The ranges of HCN reported in this study are comparable to those reported in earlier studies [36,37,38]. Based on the 1 to 9 scale, the genetic component of the variation (V_g_) was 0.48, which is higher than that of (V_g_) = 0.21 reported in African germplasm from the International Institute for Tropical Agriculture (IITA), Nigeria, and much lower than that in Colombian, (V_g_) = 1.58, and Brazilian germplasm, (V_g_) = 2.59 [38]. The higher genetic variation for HCN in Ugandan germplasm compared to IITA’s can be attributed to the deliberate inclusion of bitter landraces in the study, i.e., Tongolo, Quinine, Nyamatia, Kazimwenge, and Edwarat material, whereas the high genetic variance reported for Brazilian and Colombian germplasm is explained by the fact that Latin America, particularly Brazil, is the center of diversity for cassava [38]. Given the large genetic variability for HCN reported for Latin American cassava clones, African cassava breeding programs targeting low-HCN product profiles can enhance diversity in their populations through germplasm exchange with their Latin American counterparts. Germplasm exchange could also be beneficial for other traits like fresh root yield, in terms of which some African cassava breeding programs are not making appreciable genetic gains [39].

A clear understanding of trait heritability informs strategies to drive genetic gains [40,41] as heritability estimates are critical to selection decisions [42]. Broad sense heritability (H^2^) estimates were low to moderate across locations (H^2^ = 0.14–0.32). This could be explained by the large effect of environmental factors on HCN phenotypic expression. Furthermore, for all phenotyping methods, the residual variance was high, suggesting that better heritability estimates can be obtained with further optimization of the methods. These H^2^ estimates are, however, comparable to estimates previously reported for West African germplasm, H^2^ = 0.27 [38] and H^2^ = 0.18 [37], but are much lower than the estimate (H^2^ = 0.76–0.82) reported for Brazilian germplasm by the same authors using the 1 to 9 scale. 

Diversity in terms of the presence of bitter clones is maintained within African populations by farmers who selectively grow bitter varieties for various reasons [20,28,43]. In Ugandan communities where communal livestock grazing is carried out, it is common practice for farmers to plant guard rows of bitter cassava landraces around their fields of the sweet cassava crop to “protect them from damage by livestock”. 

Whereas Torres et al. [37] suggested three categorizations of HCN on the 1 to 9 scale, sweet (1–4), intermediate (4.1–5), and bitter (5.1–9), these categories are contestable given that the levels of taste were neither tested by a trained sensory panel nor validated by a consumer panel. Thus, there is no evidence to suggest that a genotype with a score of 5.1 or 6.0 will be perceived as bitter by the consumer. Rather, we suggest four categories that could have practical implications for cassava breeding: low HCN (1–4.0), corresponding to 0–100 ppm; intermediate HCN (4.1–5.9), corresponding to 101–200 ppm; high HCN (6.0–7.0), corresponding to 201–400 ppm; and very high HCN (7.1–9.0), corresponding to 401–900 ppm.

Thus, for breeding efforts targeting boiled products (associated with minimal processing), an average score of 1–4 is desirable, but one of less than 6.0 is acceptable. For the fresh root chewing product profile (common in Tanzania), which is associated with no processing at all, the cut-off for selection should be at 4.0. This categorization is somewhat in alignment with that of Zhong et al. [44], who recommended a maximum residue limit (MRL) of 200 ppm for cyanogenic glycosides in cassava for food. For product profiles involving extensive processing like drying, milling, and fermentation, the cut off may be relaxed to include clone scores averaging 6.0–7.0 given that significant amounts of cyanogens are lost during processing [45,46,47]. However, when initial fresh root HCN concentrations are very high, traditional processing methods may not be sufficient to detoxify the roots [45], resulting in fatalities [15,16,48]. Depending on the target product profile, different cassava breeding programs may have different cut-offs, considering the efficiency of traditional processing methods available and local and international regulations in terms of acceptable maximum residual levels of cyanide in cassava food and processed products.

### 3.2. Effect of the Environment on Fresh Root HCN Content

Fresh cassava root HCN content is known to vary even within roots of the same variety [10,49]. These variations can in part be accounted for by differences in genotypes, climatic factors, and soils [12,18,50,51]. The highest mean HCN in each location was recorded in Arua (7.7) and Serere (7.58). These locations also had the highest temperatures recorded during the crop growing period (23.9 K and 22.99 K, respectively) and the least precipitation and lowest relative humidity after 18 h. This is consistent with observations by the authors of [31,52], who reported that cyanide in cassava roots tended to increase in periods of drought and/or dry weather. 

The higher cassava root HCN levels recorded in Arua and Serere could be explained by the effect of temperature on the crop’s physiology. Cyanide in cassava is produced from the breakdown of linamarine and lotaustraline, a process catalyzed by the hydroxynitrile lyase (HNL) enzyme, which is in the leaves and stems but not in the roots [53]. Increases in temperature increase the rate of transpiration and sap flow in the plant, but could also increase the rate of HNL enzyme activity, thus elevating cassava root cyanide levels [53]. Furthermore, temperature might influence the rate of cyanide detoxification in cassava roots via the β–cyanoaline pathway, which coverts cyanide into the amino acid asparagine. This reaction is catalyzed by the β-cyanoaline synthase (CAS) enzyme, which is sensitive to heat denaturation; therefore, its activity could be suppressed at high temperature [53].

Given the projected 1.2–4.4 °C increase in temperature on the African continent between the year 2030 and 2050 [54], it is essential that cassava varieties that are high-yielding, low in HCN, and stable across environments be developed. However, variations in temperature and relative humidity alone may not fully explain the effect of the environment on HCN phenotypic expression. In Tanzania, farmers claim that low-fertility soils, and red clayey and sandy soils have a tendency to produce bitter cassava roots [30]. The effect of climatic factors and soil physical (texture) and chemical composition (pH and mineral composition) ought to be studied simultaneously across seasons and locations for more compelling observations.

BLUPS offer excellent predictive ability to guide selection decisions [55,56,57]. Since our interest is in developing low-HCN varieties, clones with low BLUP values are preferred. However, the threshold of acceptability (selection for advancement) will depend on the breeder’s target product profile and the selection intensity. Five of the bitter landraces were featured among the top ten high-HCN clones, while one (Edwarat) was surprisingly ranked among the top ten low-HCN clones under two of the methods. This clone is a landrace in a semi-arid environment in Eastern Uganda, where it is said to be always bitter; thus, its name “Edwarat”, which means “the bitter one”. This underscores the impact of environmental factors on fresh root HCN content. Cassava genotypes that are high-yielding and low in HCN across several environments are highly desirable [50]. 

### 3.3. Accuracy of HCN Phenotyping Methods

The 1 to 9 scale (CV = 15.6%, H^2^ = 0.26) is a more accurate and reproducible method compared with either the spectrophotometric method (CV = 24.6%, H^2^ = 0.14) or the 0 to 800 scale (CV = 38.5%, H^2^ = 0.32). Thus, whereas the spectrophotometric method is more accurate than the 0 to 800 scale, it is less reproducible. The 0 to 800 scale seems to be accurate for low HCN scores up to 300 ppm but is inaccurate for higher scores. The spectrophotometric method on the other hand seems less accurate for the low values (less than 400 ppm) but accurate for the high values. The large residuals seen in the 0 to 800 scale could be due to the difficulty in distinguishing picrate paper colors and assigning the correct corresponding scores, an inherent problem associated with color scales [58]. Furthermore, residuals observed when using the spectrophotometer could possibly be exacerbated by human error in time intervals when colored picrate papers are eluted in water, especially when large sample numbers must be read on the spectrophotometer in a single batch. For the 1 to 9 scale, residuals were equally spread out across the range of predicted values, suggesting constant variance in residuals across the scale. The moderate to high coefficients of determination between the methods (R^2^ = 0.57–0.85) suggest that one can predict phenotypes of one method using another with a high degree of confidence, since the methods yield comparable results.

For selection purposes, the 1 to 9 scale and spectrophotometric method outperformed the 0 to 800 scale as between them, they consistently selected 9 out of the top 10 low-HCN clones. Given the extra requirements in sample preparation, machinery, and power, which make the spectrophotometric protocol an error-prone, expensive, and lower-throughput alternative, the more accurate 1 to 9 scale should be the method of choice for routine phenotyping in cassava breeding programs. At late-stage breeding trials when the population size is small and exact fresh root HCN content is required to support variety release applications, the spectrophotometric method can be deployed. However, given the inherent difficulty in fresh cassava root HCN phenotyping, there is need to develop reproducible, accurate, and high-throughput phenotyping protocols for HCN.

Near-infrared spectroscopy (NIRS) is one of such technologies that has been adopted by major cassava breeding programs in Uganda and Nigeria for cassava root quality phenotyping [58,59,60] and has recently been shown to separate low- and high-HCN clones with high accuracy [61]. Thus, cassava breeders targeting low-HCN cassava varieties may find the concept of phenomic selection, as recently described by Robert et al. [62], more appealing compared with genome-based tools like marker-assisted selection (MAS) given the phenotypic plasticity of the trait. Acyanogenic cassava can be produced via genetic transformation [63]. However, in many African jurisdictions, as is the case in, Uganda, transgenics still faces stiff resistance from anti-transgenics lobbyist groups, and would-be-breakthrough research [64,65] is largely restricted to laboratory and confined field trials. Thus, conventional breeding for low-cyanogenic cassava remains the best avenue to safeguard the public from potential harmful effects of dietary HCN consumption.

## 4. Materials and Methods

### 4.1. Description of the Study Area

Variability for HCN was studied across four locations, The National Crops Resources Research Institute (NaCRRI), central Uganda; the National Semi-arid Resources Research Institute (NaSARRI), Serere in eastern Uganda; the Abi Zonal Agricultural Research Institute (Abi ZARDI), Arua, northern Uganda; and Tororo in eastern Uganda. NaCRRI is located at longitude 32.62717, latitude 0.521526, and 1134 m above sea level, characterized by a bi-modal rainfall pattern with an average annual rainfall value of 1325 mm and annual average temperature of 21 °C. The soils are sandy loams. NaSARRI is located at longitude 33.54901 and latitude 1.499403 with an elevation of 1104 m above sea level. The area is semi-arid with average annual temperatures of 26.5 °C, and a mean annual rainfall value of of 900 mm. The soils are light sandy loams. Abi ZARDI is located at longitude 30.95 and latitude 3.08333 at an altitude of 1109 m above sea level, experiences a bi-modal rainfall pattern with an average annual rainfall value of 1250 mm and average annual temperature of 24 °C. The soils are sandy loam soils. Finally, Tororo is located at longitude 34.0223 and latitude 0.769 with an elevation of 1278 m above sea level. The mean annual temperature is 21 °C. The area is characterized by light sandy soils and a bi-modal rainfall pattern with a mean annual rainfall value of 1450 mm.

### 4.2. Description of Study Materials and Field Trial Establishment

A set of 64 clones were used in this study. These consisted of eight bitter landraces collected from farmers’ fields across the country and 56 advanced breeding clones. These clones were selections from cycle two of the genomic selection population [66]. At each location, clones were established in the field in a randomized complete block design with two replicates. Each plot consisted of five rows of plants with a spacing of 1 m× 1 m, with 2 m alleys between plots. Plots were kept weed-free via hand weeding. No fertilizers or supplemental irrigation were applied. Further details of the trials and study materials can be found in the online open access data repository Cassavabase at www.cassavabase.org and in the Supplementary Table S1.

### 4.3. Sample Selection, Preparation, and Data Collection

#### 4.3.1. Sample Selection

At harvest (12 months after planting), three middle rows of each plot were uprooted and all harvested roots were pooled together. Three uniformly sized roots were then selected and taken to the laboratory for analysis. At the laboratory, roots were washed under running water to remove soil and debris and then dried with a towel.

#### 4.3.2. Sample Preparation

For each root, the distal and proximal portions were sliced off with a kitchen knife and discarded, leaving a 10 cm middle portion. This portion was peeled with a kitchen knife and, the parenchyma were grated with a kitchen grater. The grated slices per root were wrapped together, and a portion was immediately weighed into a falcon tube containing 5 mL of pH 8 phosphate buffer. A fresh picrate paper strip (1 cm wide, 3 cm long) prepared by dipping Whatman 1 filter paper (Whatman International Limited) in a solution of picric acid (0.5% *w*/*v*) in 2.5% (*w*/*v*) sodium carbonate and allowing the paper to dry at room temperature was immediately suspended above the falcon tube, and the tube was tightly stoppered. The tubes were kept in the dark for 16 h at room temperature. 

### 4.4. Data Collection

#### 4.4.1. Measurement of HCN

Three assessments for HCN were undertaken. Firstly, scores were taken for the final color of the picrate paper strip on a 1 to 9 scale, with 1 and 9 representing the extremes of low and high HCN content, respectively [34]. Secondly, scores of the picrate paper were also taken using the 0–800 parts per million (ppm) scale, with 0 ppm and 800 ppm representing extremes of low and high HCN content, respectively [8]. Thirdly, the scored picrate paper was eluted in 3 milliliters of distilled water for 30 min, and the absorbance of the resulting colored solution was read on a spectrophotometer (Jenway 6305 UV/Visible Spectrophotometer) at 510 nm against a similarly prepared blank but whose picrate paper was not exposed to the sample. To obtain the final HCN value in parts per million, the absorbance value of the sample was multiplied by 396 [32]. 

#### 4.4.2. Weather Data

Weather data were obtained for the study sites from the AgERA5 database [67] using the R package *ag5Tools* [68]. The AgERA5 dataset provides daily surface meteorological data from 1979 at a resolution of 0.1° [69]. Thus, daily weather data were extracted for the period September 2020 to September 2021 for the parameters 24 h temperature (K), relative humidity after 12 h (%), relative humidity after 18 h (%), and precipitation (mm/day). 

### 4.5. Data Analysis

For each of the three HCN assessment methods, an independent dataset was assembled. Data analysis was conducted in the R statistical package. An analysis of variance was conducted to test for the significance of the observed differences in the means of clones and in the locations, and for the interaction of genotypes with location (GEI) at a 0.05 alpha level. The means of clones and locations were compared using Tukey’s honestly significant difference test using the *HSD.test* function of the *Agricolae* package at a 0.05 alpha level. Broad sense heritability was computed based on single-site analysis and on a combination of datasets from all the sites. The *lmer* function of the R statistical package was used to fit the following mixed linear model:

y=Xβ+Zu+ε

where y is the response vector of HCN for a given location, β is the vector of fixed effects, u is the vector of random genetic effects with design matrix Z (relating trait values to genotype, environment, and genotype–environment interaction), and ε is the error (residual). Variance components were then extracted and used for the estimation of broad sense heritability (H^2^). Within locations (a single trial), broad sense heritability was estimated as follows:

$$H^{2}=\frac{V_{g}}{V_{g}+V_{e}}$$

where H^2^ is the broad sense heritability, V_g_ is the genotypic variance, and V_e_ is the error (residual) variance. Across locations (trials), broad sense heritability was estimated as follows:

$$H^{2}=\frac{V_{g}}{V_{g}+V_{ge}+V_{e}}$$

where $V_{g}$ is the genotypic variance, V_e_ is the error (residual) variance, and $V_{ge}$ is the variance due to the genotype–environment interaction.

The ranking of clones in accordance with HCN content was conducted using the best unbiased linear predictors (BLUPS), which were computed using the *ranef* function in R. Variance components, heritability and BLUPS were computed separately for each HCN phenotyping method. The Kruskal–Wallis test was performed to test the hypothesis that the test environments did not experience different weather conditions, and results were plotted using the *ggpubr* package. Correlation and regression analyses were performed to investigate the relationship between the HCN phenotyping methods using the packages *ggpubr* and *ggpmisc* in R.

## 5. Conclusions

Based on our results, there is appreciable genetic diversity for HCN in locally available Ugandan cassava germplasm. Broad sense heritability for HCN within the study germplasm was low to moderate, and the 1 to 9 scale is more accurate and more reproducible than either the 0 to 800 ppm scale or spectrophotometric methods. Furthermore, average daily temperature and relative humidity during crop growth influence fresh cassava root HCN phenotypic expression. This information provides frameworks for undertaking sustainable cassava breeding that is mindful of acceptable thresholds for HCN in developed products and the phenotypic plasticity of the trait.

## Figures and Tables

**Table 1:** Analysis of variance for fresh cassava root HCN content across locations.

		Mean Square		
Source of Variation	Degrees of Freedom	1–9_Scale	HCN_0–800_Scale	HCN_Spec
Clone	63	5.069 ***	130,338 ***	57,706 ***
Location	3	127.548 ***	3,384,185 ***	1,127,633 ***
Replication	1	1.133	33,359	670,202 ***
Accession: Location	149	1.571 **	33,731 *	27,349 ***
Residuals	147	1.085	27,283	14,280

Significance codes: * < 0.05, ** < 0.01, and *** < 0.001; 1–9_Scale = HCN scored on 1 to 9 categorical scale, HCN_Spec = HCN values in parts per million computed from spectrophotometric readings, and HCN_0–800_Scale = HCN scored on a 0 to 800 parts per million categorical scale.

**Table 2:** Summary of phenotypic variation, variance components, and broad sense heritability for HCN in fresh cassava roots across locations and HCN phenotyping methods.

Location	HCN_1–9_Scale	SD	H^2^	HCN_0–800_Scale	SD	H^2^	HCN_Spec	SD	H^2^
Arua	7.70 ^a^	1.26	0.46	614.24 ^a^	231.72	0.64	601.85 ^a^	136.03	0.50
Serere	7.58 ^a^	0.97	0.54	595.68 ^a^	205.842	0.24	608.19 ^a^	66.16	0.22
Namulonge	5.45 ^b^	1.77	0.50	277.48 ^b^	216.6	0.52	407.0 ^b^	231.15	0.56
Tororo	5.75 ^b^	1.34	0.48	263.03 ^b^	204.29	0.56	399.39 ^b^	181.92	0.40
CV (%)	15.6			38.50			24.60		
V_g_	0.48			13,990			3596		
V_ge_	0.35			4943			7748		
V_Res_	1.04			24,897			14,093		
H^2^	0.26			0.32			0.14		

Means followed by the same letter are not significantly different at the 5% alpha level. HCN = hydrogen cyanide; SD = standard deviation; Spec = spectrophotometer; CV = coefficient of variation; % = percentage; V_g_ = genotypic variance; V_ge_ = genotype–environment variance; V_Res_ = residual variance; H^2^ = broad sense heritability.

**Table 3:** Ranking of selected clones from highest to lowest HCN content using BLUP values for the three HCN evaluation methods.

1–9 Scale			0–800 ppm Scale			HCN_Spec		
Accession	BLUP	Classification	Accession	BLUP	Classification	Accession	BLUP	Classification
TONGOLO	1.306	High	TONGOLO	257.776	High	KAZIMWENGE	63.186	High
NYAMATIA	1.085	High	NYAMATIA	225.384	High	UG16F300P068	59.250	High
KAZIMWENGE	0.883	High	QUININE	181.413	High	TONGOLO	56.682	High
QUININE	0.810	High	KAZIMWENGE	177.962	High	UG16F158P004	55.176	High
UG16F303P006	0.785	High	UG16F303P006	124.465	High	UG16F063P006	54.696	High
UG16F290P332	0.768	High	UG16F063P006	120.565	High	NYAMATIA	53.768	High
UG16F303P009	0.758	High	UG16F303P009	114.728	High	UG16F294P011	44.224	High
UG16F158P004	0.734	High	UG16F158P004	103.961	High	UG16F293P151	42.927	High
UG16F294P011	0.707	High	UG16F300P068	102.232	High	UG16F077P002	42.798	High
UG16F063P006	0.672	High	UG16F159P001	91.997	High	QUININE	36.430	High
EDWARAT	−0.628	Low	UG16F290P073	−102.508	Low	EDWARAT	−51.843	Low
UG16F290P128	−0.743	Low	UG16F290P040	−107.260	Low	UG16F290P073	−52.257	Low
UG16F293P082	−0.753	Low	UG16F314P005	−107.724	Low	UG16F290P128	−52.338	Low
UG16F303P005	−0.779	Low	UG110017	−109.308	Low	UG16F293P066	−52.881	Low
UG16F290P295	−0.882	Low	UG16F303P005	−119.431	Low	UG16F293P169	−54.483	Low
UG16F293P169	−1.039	Low	UG16F057P001	−122.378	Low	UG16F293P082	−56.428	Low
UG16F057P001	−1.090	Low	UG16F290P295	−125.102	Low	UG16F057P001	−70.226	Low
UG16F001P013	−1.262	Low	UG16F293P169	−137.138	Low	UG16F001P013	−71.706	Low
UG16F293P066	−1.361	Low	UG16F001P013	−189.676	Low	UG16F290P295	−81.562	Low
UG16F290P075	−1.574	Low	UG16F290P075	−226.642	Low	UG16F290P075	−101.391	Low

HCN = hydrogen cyanide, Spec = spectrophotometer, BLUP = best linear unbiased predictor. Only the top-ten high-HCN and top-ten low-HCN clones are shown.

## Data Availability

Data are available in an online open-access repository (Cassavabase) via the link: https://cassavabase.org/ftp/manuscripts/Kanaabi_et_al_2024/ (accessed on 12 February 2024).

## Supplementary Materials

The following supporting information can be downloaded at: https://www.mdpi.com/article/10.3390/plants13091186/s1, Table S1: Description of study materials.
